# Quantifying unpredictability: A multiple-model approach based on satellite imagery data from Mediterranean ponds

**DOI:** 10.1371/journal.pone.0187958

**Published:** 2017-11-09

**Authors:** Lluis Franch-Gras, Eduardo Moisés García-Roger, Belen Franch, María José Carmona, Manuel Serra

**Affiliations:** 1 Institut Cavanilles de Biodiversitat i Biologia Evolutiva, Universitat de València, Valencia, Spain; 2 NASA Goddard Space Flight Center, Greenbelt, MD, United States of America; 3 Department of Geographical Sciences, University of Maryland, College Park, MD, United States of America; University of Mexico, UNITED STATES

## Abstract

Fluctuations in environmental parameters are increasingly being recognized as essential features of any habitat. The quantification of whether environmental fluctuations are prevalently predictable or unpredictable is remarkably relevant to understanding the evolutionary responses of organisms. However, when characterizing the relevant features of natural habitats, ecologists typically face two problems: (1) gathering long-term data and (2) handling the hard-won data. This paper takes advantage of the free access to long-term recordings of remote sensing data (27 years, Landsat TM/ETM+) to assess a set of environmental models for estimating environmental predictability. The case study included 20 Mediterranean saline ponds and lakes, and the focal variable was the water-surface area. This study first aimed to produce a method for accurately estimating the water-surface area from satellite images. Saline ponds can develop salt-crusted areas that make it difficult to distinguish between soil and water. This challenge was addressed using a novel pipeline that combines band ratio water indices and the short near-infrared band as a salt filter. The study then extracted the predictable and unpredictable components of variation in the water-surface area. Two different approaches, each showing variations in the parameters, were used to obtain the stochastic variation around a regular pattern with the objective of dissecting the effect of assumptions on predictability estimations. The first approach, which is based on Colwell’s predictability metrics, transforms the focal variable into a nominal one. The resulting discrete categories define the relevant variations in the water-surface area. In the second approach, we introduced General Additive Model (GAM) fitting as a new metric for quantifying predictability. Both approaches produced a wide range of predictability for the studied ponds. Some model assumptions–which are considered very different *a priori*–had minor effects, whereas others produced predictability estimations that showed some degree of divergence. We hypothesize that these diverging estimations of predictability reflect the effect of fluctuations on different types of organisms. The fluctuation analysis described in this manuscript is applicable to a wide variety of systems, including both aquatic and non-aquatic systems, and will be valuable for quantifying and characterizing predictability, which is essential within the expected global increase in the unpredictability of environmental fluctuations. We advocate that *a priori* information for organisms of interest should be used to select the most suitable metrics for estimating predictability, and we provide some guidelines for this approach.

## 2. Introduction

Fluctuations in environmental parameters and their potentially associated unpredictability are increasingly being recognized as essential features of any habitat [1] because they are expected to influence the performance of the inhabiting organisms and affect upper levels of ecological organization [2]. Because human activity often increases environmental fluctuations, their analysis provides an applied interest [3–5]. Indeed, ascertaining how organisms respond to environmental fluctuations is fundamental in biology [6,7].

From the point of view of an organism, predictability is related to the organism’s ability to anticipate and adjust to a future environmental condition and thus involves a time scale [8,9]. For instance, annual fluctuations in temperature might be experienced very differently by short-living invertebrates compared with long-living vertebrates [10]. Any habitat shows some constancy and some variations in its features, and the focal habitat feature can consistently be decomposed into three components: constancy, predictable–periodic–fluctuations, and unpredictable fluctuations. The relative importance of these components is expected to produce diverging adaptive responses in organisms [11]. Thus, to quantify whether environmental fluctuations are prevalently predictable or unpredictable is highly relevant for understanding evolutionary responses [2] and for testing ecological and evolutionary hypotheses.

The characterization of fluctuations is a complex problem that requires a robust methodology because it cannot be based on mean values but rather on variances. Moreover, this characterization needs predefined assumptions regarding the relevant time-scales that can be associated with predictability, which cannot always be defined in a straightforward manner. Furthermore, one of the most important constraints is the need of long time-series data. Thus, (1) a methodology for the acquisition of long-enough time series is required, and, (2) an appropriate metric then has to be assumed and assessed to characterize the focal habitats.

Remote sensing technology has been highly developed in the last century [12], but its use in several research areas has not reached yet its full potential, as is the case in ecology [13]. Several studies have shown that this technology can offer solutions to a wide variety of problems in nearly all fields of environmental research [14–16]. Long time series are costly to obtain and are consequently scarce [17]. In this regard, the information gathered by several satellites in recent years can provide long time-series data, such as the Landsat satellite series, which ranges from 1972 to the present and has moderate spatial and intermediate temporal resolutions [18]. Since 2008, these scenes are freely available from the United States Geological Survey (USGS), which allows the scientific community to gather a great deal of information free of cost [19].

Several indices have been developed to estimate the degree of predictability from time-series data. Basically, these indices decompose the time series based on periodic and stochastic variation [20] and associate these with predictable and unpredictable fluctuations, respectively. For nominal data, such as the presence/absence of water, Colwell [21] proposed a predictability index based on information theory [22] that has been mainly used in streams and rivers [23]. In contrast, indices based on spectral analysis, such as the Fourier transform and asymmetric eigenvector maps (AEM) [24, 25], have been used for continuous data [26]. However, these continuous-metric methods are very sensitive to gaps in the time series; thus, Colwell’s method might be preferable despite the drawback of discretizing a quantitative variable.

In this study, a group of Mediterranean shallow ponds and lakes were considered the case study, and the water-surface area (hereafter *A*; [27]) was considered as focal habitat feature. *A* is considered an ecologically relevant factor in lentic water bodies [28] and is correlated with the average lake depth, and both of these measurements determine the habitat size for aquatic organisms. The habitat size affects the ‘colonization vs. extinction’ balance and the habitat heterogeneity [29]. Fluctuations in *A* have multiple effects on the organisms above and below the waterline, including both aquatic flora (e.g., macrophytes [30]) and fauna (e.g., fish [31] and zooplankton [32]). These fluctuations can strongly affect the habitat conditions through variations in the physicochemical parameters (e.g., temperature, light, nutrient or solute concentration [33,34]). The solute concentration is particularly relevant in saline ponds and lakes, which are a significant, geographically widespread part of the world’s inland aquatic ecosystems [35, 36]. However, despite their importance, remote sensing studies in saline water bodies are scarce and have mostly been based on large lakes [37–39]. Overall, band ratios are typically used when detecting water bodies (e.g., [40, 41]), but are rarely used for saline ponds. In contrast, infrared bands have been previously used for the assessment of *A* in saline ponds, but these are not accompanied by band ratio indices [42, 43].

Water bodies in the Mediterranean region are a good case study because they are expected to cover wide ranges of predictabilty. Some of these water bodies are almost permanent, whereas others are characterized by strong seasonality and temporal unpredictability at several time scales [44]. Both confined and unconfined organisms in non-permanent ponds are expected to undergo adaptation and to be strongly reliant on patterns of pond inundation (i.e., *A* variation). Waterfowls exhibit migratory patterns and many short-lived animals undergo lifecycles at an annual or shorter time scale. Accordingly, ecological unpredictability in these systems can be conceived as departures from an average seasonal variation, i.e., the main source of environmental unpredictability is the inter-annual variation of the within-year variation.

In this study, we used time-series remote sensing data to assess a set of models for the estimation of environmental predictability. Our study is divided in two parts. The first part of this manuscript proposes a procedure to estimate *A* and assesses this procedure using a set of Mediterranean saline water bodies that can develop salt crusts by evaporation. In the second part, a set of metrics for estimating stochastic variations in *A* are elaborated and compared to evaluate their effects on the assessment of environmental predictability. Specifically, in the first part, *A* was quantified using 27 years of Landsat 5/7 scenes. A salt crust is formed in some of the evaluated ponds when water is evaporated, and this salt crust can make it difficult to distinguish between soil and water. This challenge was addressed through the development of a novel approach that combines the sequential use of band ratio water indices and the short near-infrared band as a salt filter. In this section of the manuscript, a hypothesis regarding the reliability of estimating *A* from satellite scenes underlies our work. The accuracy of these estimations was assessed through the inspection of aerial (non-satellite) images and qualitative field data. In the second part, the predictable and unpredictable components of the variation in *A* over the 27-year time series for each pond were extracted. Two different approaches, which showed differences in their parameters, were used to obtain the stochastic variation around a regular pattern with the objective of dissecting the effect of assumptions on predictability estimations. The first approach, which is based on Colwell’s predictability metrics, transforms the focal variable into a nominal one. The resulting discrete categories define which variations in the water-surface area are considered relevant. For the second approach, we developed a novel approach that parallels the method developed by Colwell but is based on regression models. The similarity and divergence of the different predictability indices were analysed to determine both their capability to embrace a wide predictability range and their sensitivity to the initial assumptions. When the indices produce diverging results, we propose that this divergence is related to the fact that the unpredictability of a fluctuation pattern varies depending on the organism of interest.

## 3. Materials and methods

### 3.1. Study area

**Fig 1:**
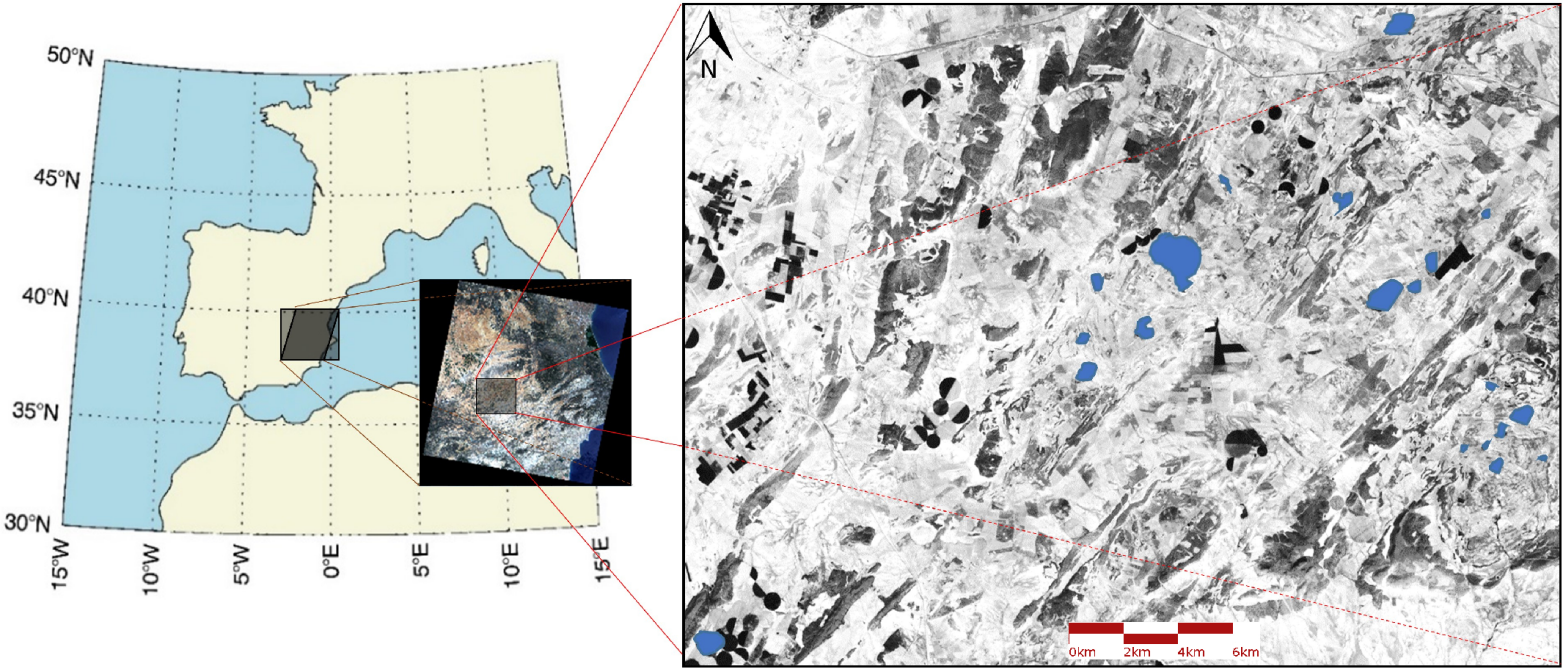
Location of the study region (38°55.4’ to 38°41.803’N and 1°47.32’ to 1°24.26’W) and Landsat 5 scene showing the area where the ponds (highlighted in blue) included in this study are located.

The study region is located in the eastern region of the Iberian Peninsula (Fig 1), an endorheic area of approximately 800 km^2^ with more than 100 ponds, and 20 of these ponds were included in this study (Table 1). The ponds included are shallow (depth of approximately 1 m) and brackish (salinity: 6.5–40 g/L) and showed different mean areas (0.00013–1.19 km^2^). Precipitation is the main water inflow, but some ponds are also connected to groundwater [45].

**Table 1:** Studied ponds sorted by decreasing mean water-surface area ($\bar{A}$)^a^.

Pond name	Pond acronym	Pond location	$\bar{A}\pm S.E.$ (m^2^)	Hydroperiod ^a^^,^^b^
**Pétrola**	PET	38°50'16.82"N, 1°33'49.22" W	1190000±140000	1.00
**Salobralejo**	SAL	38°54'52.11"N, 1°28'6.95"W	237000±18000	1.00
**Ontalafia**	ONT	38°43'21.23"N, 1°46'3.91"W	209000±13000	0.99
**Hoya Grande**	HYG	38°49'35.17"N, 1°28'31.17"W	141000±18000	0.69
**El Saladar**	SLD	38°47'21.72"N, 1°25'8.00"W	111000±8000	1.00
**Atalaya de los Ojicos**	ATA	38°46'20.97"N, 1°25'49.12" W	47000±3000	0.93
**Horna**	HOR	38°50'0.77"N, 1°36'3.87"W	41000±7000	0.53
**Hoya Rasa**	HYR	38°47'6.06"N, 1°25'37.56" W	40000±4000	0.87
**Casa Villora**	CVI	38°48'11.47"N, 1°36'18.10"W	36000±6000	0.48
**Hoya Redonda**	HRE	38°49'5.88"N, 1°34'49.96"W	34000±6000	0.30
**Hoya del Norte**	HYN	38°50'17.10"N, 1°27'23.08"W	32000±6000	0.43
**Hoya Chica**	HYC	38°49'46.22"N, 1°27'49.74"W	32000±4000	0.51
**La Campana**	CAM	38°51'29.06"N, 1°29'36.97" W	29000±4000	0.63
**Mojón Blanco**	BLA	38°47'49.95"N, 1°25'55.47"W	19000±1700	0.89
**Hoya del Monte**	HMT	38°50'44.87"N, 1°26'38.70"W	15800±1900	0.51
**Casa Villora2**	CVI2	38°49'1.33"N, 1°36'37.03"W	5600±1000	0.26
**Hoya de las Ánades**	HYA	38°51'44.84"N, 1°32'38.59"W	4900±900	0.22
**Hoya Yerba**	HYB	38°46'46.02"N, 1°26'6.60"W	1060±230	0.23
**Hoya Elvira**	HYE	38°46'42.13"N, 1°26'43.36"W	230±70	0.09
**Hoya Turnera**	HTU	38°46'31.19"N, 1°24'37.41"W	130±50	0.07

^a.^ Obtained from satellite data in this study

^b.^ Estimated as the average of each month’s fraction of observations with *A*>0 and cloud cover = 0.

The climate in the study area is semiarid, with a mean average annual rainfall of 343 mm and a mean temperature of 14°C (local weather station). June to September is considered the dry period, when temperatures might exceed 40°C and rainfall is scarce (typically <8 mm in July). Most precipitation occurs as heavy rains in spring (April-May) and autumn (October-December) [45].

**Fig 2:**
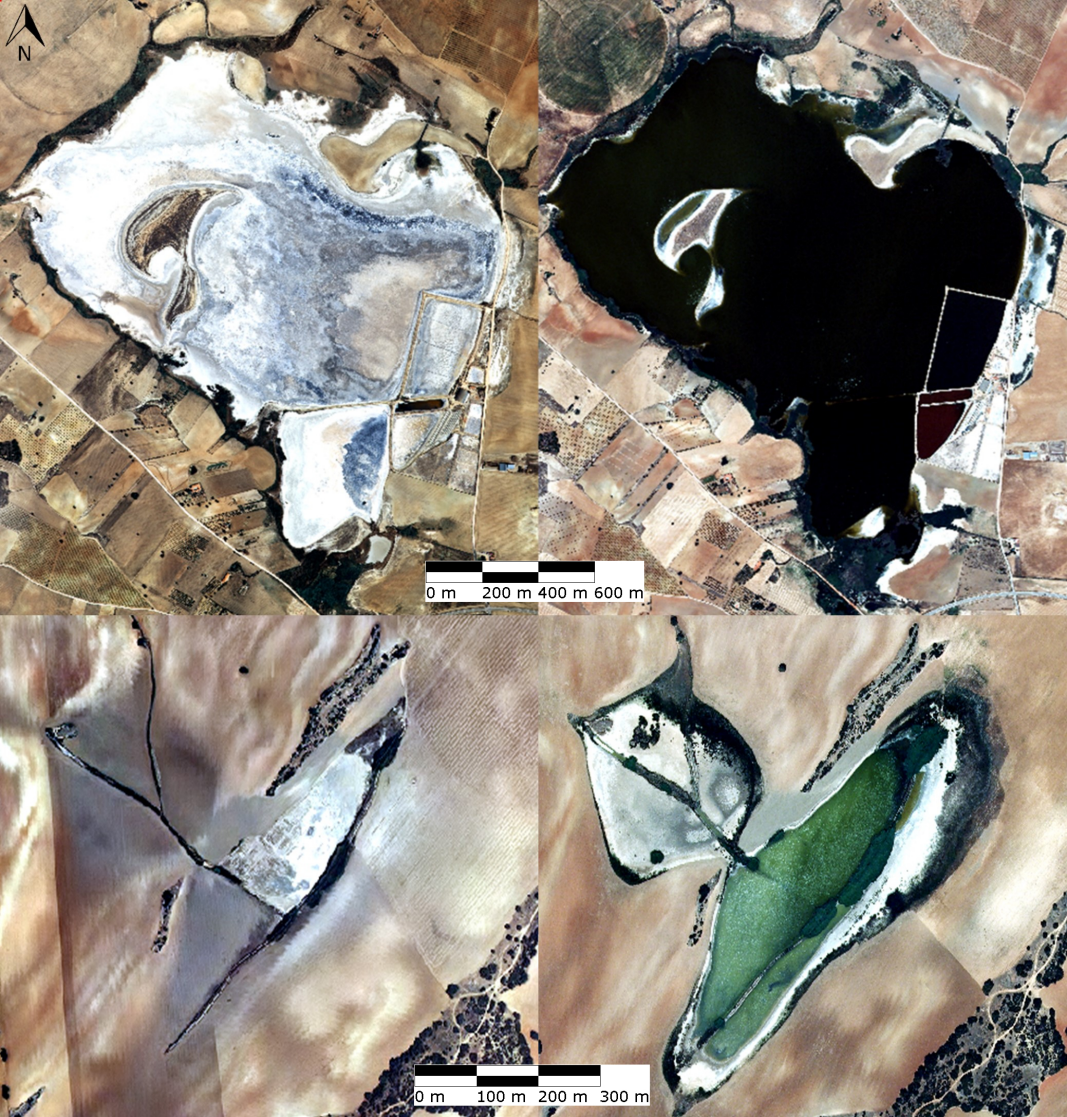
Aerial images of the ponds PET (above) and CAM (below), illustrating the variations in the water-surface area (green-black) and salt crust (white).

Episodic, *in situ* observations over the last decade have shown that some ponds can frequently dry out, develop a thick salt crust, and fill up again (Fig 2). In contrast, other ponds, were observed to be flooded for years, including through several dry seasons.

### 3.2 Satellite scene data

In this study, we used a total of 432 images over the path 199 and row 33 scene acquired by the Landsat Thematic Mapper (Landsat 5) and Enhanced Thematic Mapper Plus (Landsat 7) satellites. Among these images, 314 were provided by the European Space Agency (ESA) as the L1T product, which means that they are radiometrically and geometrically corrected [46]. The remaining 118 scenes were downloaded from the United States Geological Survey (USGS) Climate Data Record (CDR) product. This product consists of surface reflectance images, which were atmospherically corrected using the Landsat Ecosystem Disturbance Adaptive Processing System (LEDAPS) algorithm [47] and an accurate cloud mask developed in a previous study [48]. The mentioned algorithm has been implemented, tested, validated and widely distributed [49]. According to a previous study [50], the LEDAPS application to Landsat has demonstrated a performance comparable to that of the MODIS algorithm for aerosol retrieval over land [49,51] and for the surface reflectance product [52,53]. To maintain consistency between the two datasets (ESA and USGS), the ESA images were processed with the LEDAPS algorithm [54]. The resulting set of scenes cover the period 1984–2011, with a spatial resolution of 30 m and a revisit time of 16 days for each satellite [18].

### 3.3 Estimation of the water-surface area (*A*)

Water presence in a pixel was inferred from a two-condition assessment (2cA). The first condition of 2cA was addressed to differentiate areas potentially covered by water from soil. The Modified Normalized Difference Water Index (MNDWI [40]) was calculated using the reflectance from Landsat’s band 2 (Green, 0.52–0.60 μm) and Landsat’s band 5 (middle infrared band, MIR, 1.55–1.75 μm) as follows:

$$\mathrm{MNDWI}=\frac{\mathrm{Green}\text{-}\mathrm{MIR}}{\mathrm{Green}+\mathrm{MIR}}.$$ (1)

As described previously [40], the pixels with positive MNDWI values were selected as potential water-covered areas.

**Fig 3:**
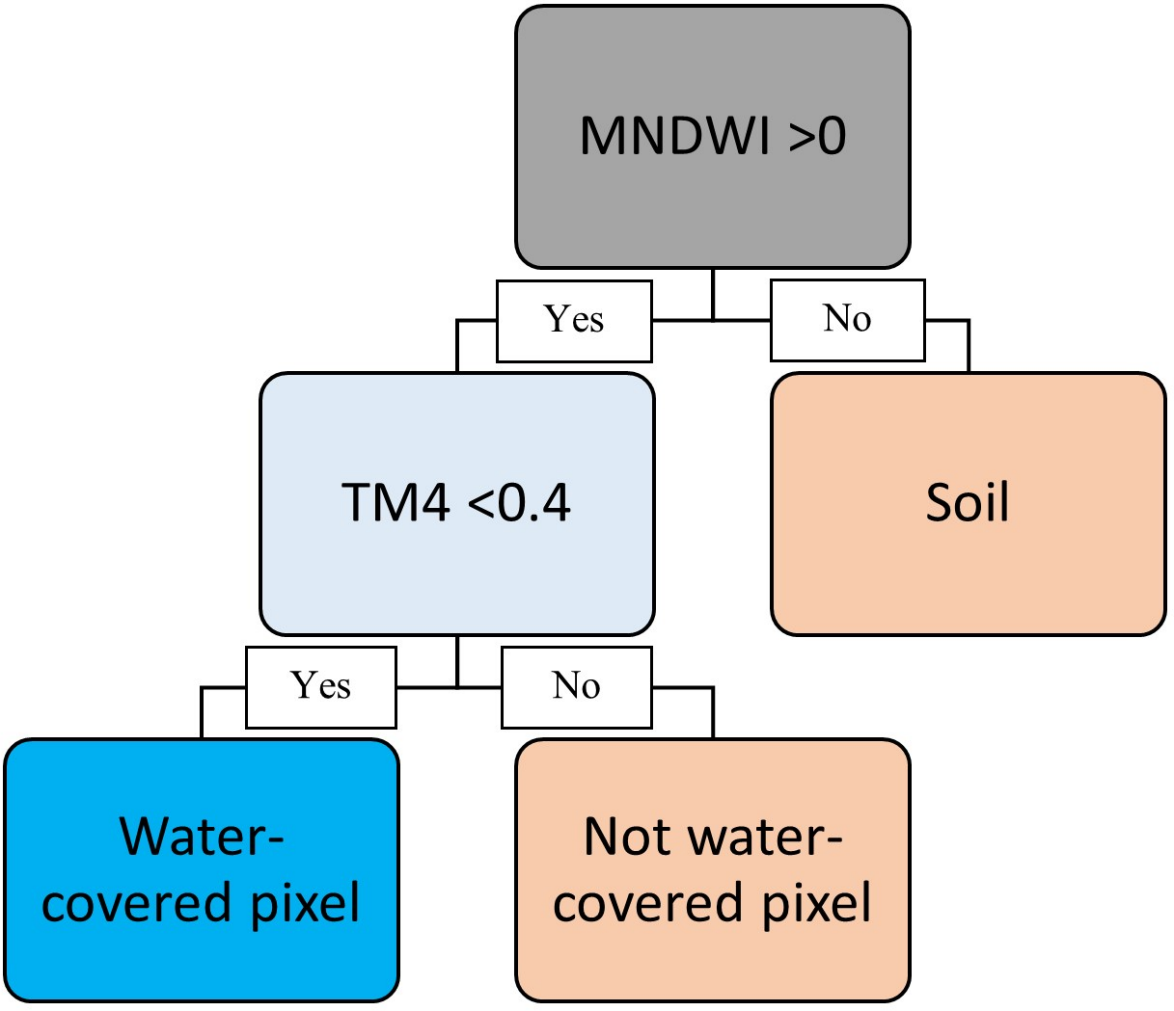
Water presence in a pixel: A two-condition assessment (2cA). The first condition differentiates potentially water-covered pixels from soil, and the second condition differentiates salt-covered from water-covered pixels.

The potentially water-covered pixels were evaluated in terms of a second condition. The salt crust left by water evaporation yielded false–positive pixels of water-covered areas. To exclude them, a second filter (salt filter) was included using a previously developed approach [42] based on the condition that band 4 (near-infrared) reflectances lower than 0.4 were classified as non-salt. However, although these authors applied the conditions to land pixels, we applied the threshold to the scenes after the first condition was assessed (Fig 3). To evaluate the adequacy of the salt filter, we compared the automatically processed results obtained after using only the first or after using both filters (2cA) through visual interpretations of aerial photographs (years 2006 and 2009; four bands; spatial resolution: 0.25–0.5 m) and qualitative field observations (presence/absence of water); S1 Table provides information for the two latter sources of validation. Satellite data analyses were performed using ENVI/IDL (Exelis Visual Information Solutions, Boulder, CO, USA).

### 3.4 Quantification of environmental predictability

For each of the ponds, the degree of predictability in the variation of *A* in the time series was estimated. Seven models with different assumptions were evaluated. Note that the term ‘model’ in this manuscript refers to a different approach to quantitatively implement *a priori* concepts to calculate the predictability indices. Model assumptions have implications on which variation of *A* is regarded as predictable. Our evaluation consisted of identifying similarities and divergences between model outputs, and determining whether the divergences are related to the capability of different organisms to predict environmental fluctuations. Five models were developed in the present study according to Colwell’s approach, and discrete categories for the range of *A* values in the time series were established. Two additional models, which constitute novel contributions of this study, followed a continuous approach, and therefore, raw observations of *A* were used. The different years in the time series were treated as replicates; thus, the seven models were applied to the within-year variation. The mean water surface area ($\bar{A}$) for each pond was computed by averaging each month’s mean *A* (excluding cloud-covered observations). The hydroperiod was estimated as the annual average of the fraction of observations with *A*> 0 in each month (excluding cloud-covered observations).

#### 3.4.1 Discrete models based on Colwell’s approach

Colwell’s predictability (*P*) index [21,22] consists of the summation of two metrics: constancy (which measures the degree in which a pond remains in the same state) and contingency (which measures the repeatability of the variation pattern). Colwell’s approach applies to nominal variables (values = states), and therefore *A* (a continuous variable) was transformed into a nominal variable. The five discrete models tested are described in Table 2.

**Table 2:** Models for predictability estimation. Statistic: parameter of the data distribution used to define the states; scaling: proportion between consecutive range windows; s_i_: i-th state (discrete); raw data: water-surface area (*A*) after image processing.

Model	Features			
Type Acronym	Excluded data	Statistic (Scaling)	Range	Definition of States
**Discrete**				
**COL_wd**^**a**^	None	-	(0, 1)	s_1_, if *A* = 0; s_2_, if *A* > 0.
**COL_Ana**^**b**^	None	Mean	(0, 1)	s_1_, if *A* < (1–0.7) · $\bar{A}$; s_2_, if (1–0.7) · $\bar{A}$ ≤ *A* ≤ (1+0.7) · $\bar{A}$; s_3_, if (1+0.7) · $\bar{A}$ < *A*.
**COL_Anw**^**c**^	Dry states	Mean	(0, 1)	As in COL_Ana
**COL_MAXlin**^**d**^	None	Maximum (Linear)	(0, 1)	s_1_, if *A* < 1/3 · *MAX*(*A*); s_2_, if 1/3 · *MAX*(*A*) ≤ *A* ≤ 2/3 · *MAX*(*A*); s_3_, if 2/3 · *MAX*(*A*) < *A*.
**COL_MAXg**^**e**^	None	Maximum (Geometric)	(0, 1)	s_1_, if *A* < ¼ · *MAX*(*A*); s_2_, if ¼ · *MAX*(*A*) ≤ *A* ≤ 2/4 · *MAX*(*A*); s_3_, if 2/4 · *MAX*(*A*) < *A*.
**Continuous**				
**GAM_a**^**f**^	None	-	(0, ∞)	Raw data
**GAM_w**^**g**^	Dry states excluded for mean computation	-	(0, ∞)	Raw data

^a^ Colwell water/dry model.

^b^ Colwell average neighborhood with all data included model.

^c^ Colwell average neighborhood with water presence model.

^d^ Colwell maximum value linear scaling model.

^e^ Colwell maximum value geometric scaling model.

^f^ GAM with mean calculated with all data included model.

^g^ GAM with mean calculated when water is detected model

The rationale for the number of states and thresholds was straightforward in some models, such as COL_wd, in which two states (presence/absence of water) were considered. The number of states in the remaining discrete models was limited to three to (1) avoid excessive proliferation of states and (2) accumulate sufficient data for each state. The thresholds between states in COL_ANa and COL_ANw were defined under the assumption that most organisms have a rather broad range of tolerance around the mean value of *A*, at which they are expected to be best adapted (being vulnerable only to extreme values); nonetheless, it also accounts for other organisms that are opportunistic for extremely high or low *A* values. In other words, they focus on separating extreme values from regular values. Consequently, in these models, the thresholds were $\bar{A}$ ± 70% $\bar{A}$, which shows a fairly wide intermediate range. Finally, in COL_MAXlin and COL_MAXg, most organisms would have a narrow tolerance range, confined to one of the proposed partitions of the maximum value. In these models, the thresholds were linear and geometric divisions of the maximum value, respectively, based on the assumption that the geometric scaling of the variation of *A* around large values has a lower effect than the same variation around small values.

In Colwell’s approach, time is also required to be a nominal variable; thus, in our models, time steps were matched to calendar months. In the months with more than one observation of *A*, the *A* value was obtained by averaging *A* observations with the lowest cloud cover. For those observations where partial cloud cover of the pond was found, the observation was only considered if the assignation of a state was not affected by the cloud covered area of the pond. Otherwise, the observation was excluded.

#### 3.4.2 Continuous models: GAM predictability

This family of models is based on the dispersion of observations in the time series around a typical intra-annual curve of the normalized *A* (hereon *A’*, normalization: $A/\bar{A}$). Because a continuous approach was used, any observation with cloud cover was excluded. The typical curve was fitted to *A’* (dependent variable) in relation to the day of the year. To avoid assuming an *a priori*, general, very constrained shape for the curve (e.g., sine function), we performed General Additive Models (GAM) with cubic splines as the smoothing function for identifying trends in the time-series data [55] using the gam function (‘MGCV’ package, [56]) in R statistical software v.2.12.1 [57].

Predictability was estimated based on the departure of the observed values from the values fitted by the GAM regression. The determination coefficient of the regression model regards constancy as a lack of determination (prediction) from the independent variable (here, time) and was thus not used. Hence, an index of predictability (P_GAM_) was developed based on the dispersion of the data with respect to the fitted model:

$$P_{\mathrm{GAM}}=\frac{1}{{\mathrm{SD}}_{\mathrm{res}}}$$ (2)

where *SD*_*res*_ is the standard deviation of the residuals of the fitted model. Our predictability index is inversely related to the coefficient of variation produced by the regression, which is defined as the mean value of the independent variable divided by *SD*_*res*_. Note that the normalization of the independent variable (*A’*) causes its mean values to approach 1, particularly when the values with *A* = 0 were not excluded.

#### 3.4.3 Relationships among predictability estimations

Pearson’s correlation coefficients were computed to evaluate the relationships among the predictability estimates from each model using (1) the 20 studied ponds and (2) 18 ponds (excluding the two most ephemeral ponds; more than 90% of time series *A* = 0; HTU and HYE) because these were expected to pose problems in regards to continuous metrics of predictability (see [20]). To facilitate interpretation of the results, a standard Unweighted Pair Group Method with Arithmetic Mean (UPGMA) cluster analysis was performed with the ‘Pvclust’ package in R [58] using a dissimilarity matrix based on the chord distance ($\sqrt{\left(2-2∙r\right)}$) of the predictability values of the 20 ponds, where *r* is Pearson’s correlation coefficient [59], and the data were resampled 10,000 times.

## 4. Results

### 4.1 Estimation of water-surface area (*A*): Assessment

**Fig 4:**
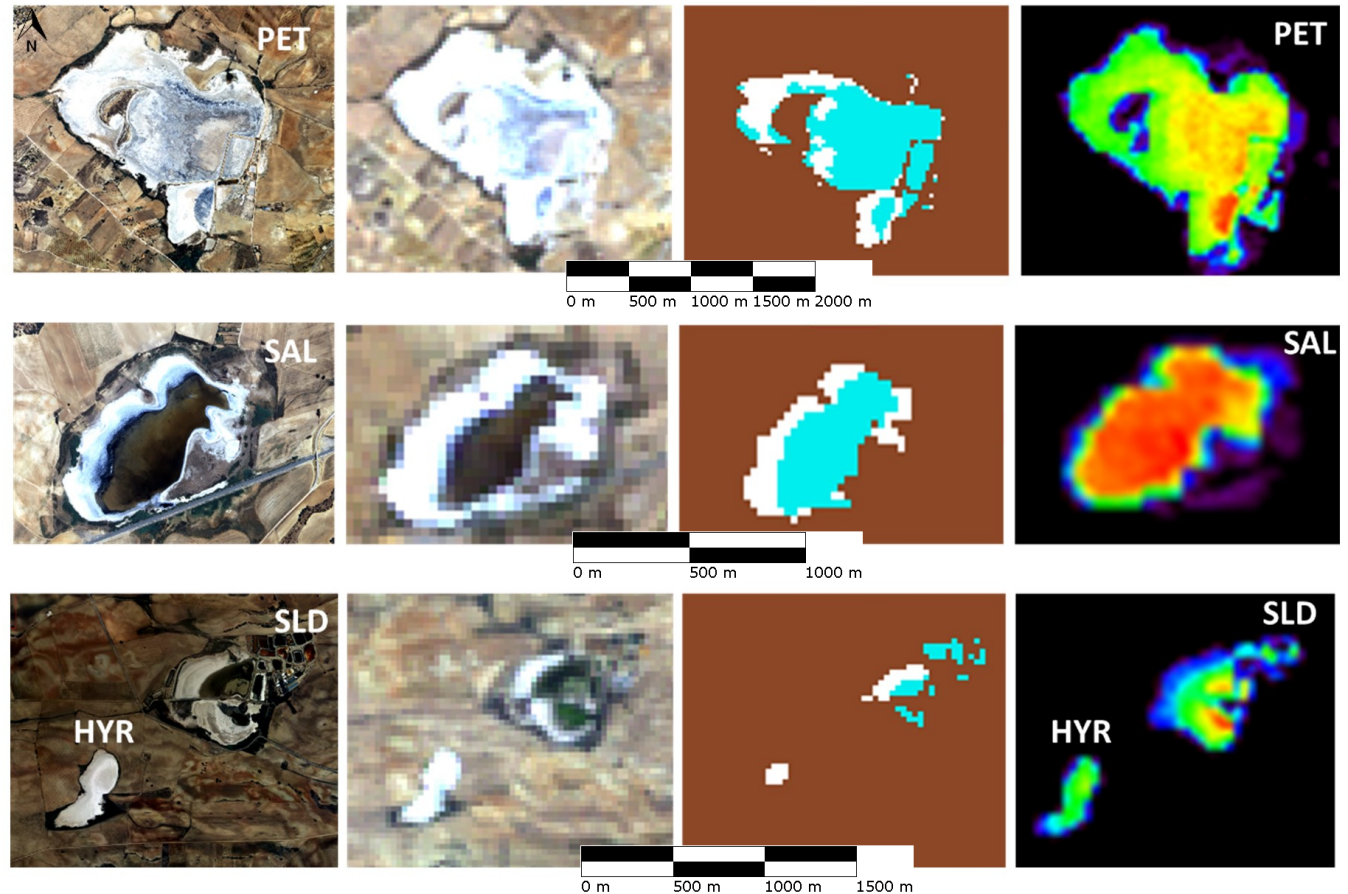
Raw and processed data for four ponds. First column: aerial scenes. Second column: Landsat 5 satellite raw scenes. Third column: results after applying the two-condition assessment to each pixel in each satellite scene. Brown pixels do not accomplish the first condition (MNDWI<0). White pixels do accomplish the first condition but not the second one (MNDWI>0 and TM4>0.4). Blue pixels do accomplish both conditions (MNDWI>0 and TM4<0.4, i.e., the pixel is considered a water-covered pixel). Fourth column: from black (0%) to red (100%), proportion of scenes for each pixel that were estimated as water-covered.

To assess the accuracy of the *A* estimations, the estimations were compared with (1) inspections of aerial images, and (2) qualitative field data (presence/absence of water in the pond). Additionally, *A* values that were visually estimated from raw scenes were compared to *A* estimations (i.e., produced by automatic processing after applying the 2cA), and both sets of estimations yielded consistent results. As a quantitative assessment, a total of 33 estimations of *A* and estimations of water-covered pixels based on aerial scenes taken close in time (maximum time for matching values corresponding to the two estimations was 18 days) were compared. Both estimations were found to be strongly correlated (*R*^2^ = 0.98, *P*-value<0.001; right panel in S1 Fig; S1 Table), and no statistically significant differences were found between them (paired *t*-test; *t* = 1.3, *d*.*f*. = 31, *P*-value = 0.2). Fig 4 shows instances of this comparison, stressing the role of applying the filter for salt crust involved in 2cA. Moreover, an estimation of *A* from satellite scenes based on only the first condition of the 2cA (i.e., without applying the salt filter) and a comparison to estimations of water-covered pixels from aerial scenes are shown in the left panel of S1 Fig. This quantitative assessment was complemented by a qualitative analysis in which *A* estimations were compared with direct qualitative field observations (presence/absence of water in the pond; *n* = 52) from previous studies [60,61] and Montero-Pau (personal communication) obtained close in time (within the same month, S1 Table). The presence/absence of water observed in the field matched in all cases (*n* = 52) with the satellite observations.

The comparison between automatically processed estimations of *A* with and without the salt filter (i.e., the second condition in the 2cA) showed that this filter causes a 7.5% (overall mean) reduction in *A*. For some of the ponds, no pixels were excluded by this condition (six ponds), but in some others the reduction can achieve up to 100% of the pixels (S2 Fig). Therefore, the second condition has different effect on *A* depending on the pond that is evaluated.

### 4.2 Estimation of the water-surface area (*A*): Historical data record

From the 27 years of monitoring, 8,640 estimations of *A* (20 ponds x 432 raw scenes) were obtained. The maximum estimated size of the ponds ranged from 5 to 2,513 pixels. The automatic application of the 2cA to raw satellite scenes yielded 4,036 cloud-free *A* data points (average per pond = 201; range 92–257). The complete time series of *A* for each pond are provided in S2 Table. For the smallest pond, *A* ranged from 0 to 4,500 m^2^, and for the largest pond, the range of this value ranged from 17,000 to 2,263,000 m^2^. In the time series of *A*, the Pearson’s correlation coefficient (*r*) between ponds ranged from -0.31 to 0.86. The annual hydroperiod ranged from 0.07 to 1.00 (Table 1). Three ponds (PET, SAL and SLD) achieved the maximum hydroperiod because these ponds never completely dried out in our data record.

### 4.3. Quantification of environmental predictability

**Fig 5:**
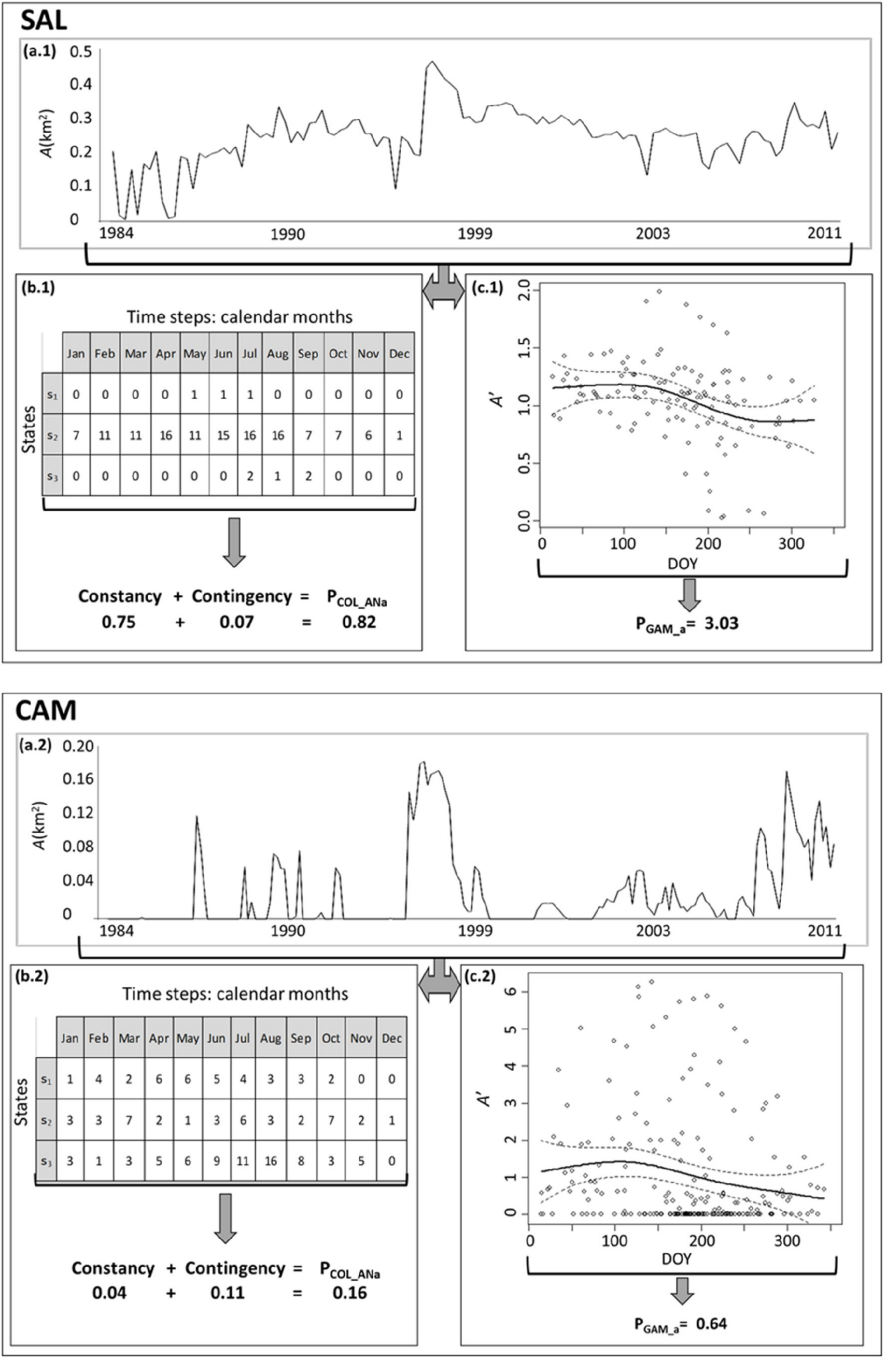
Instances outlining the data analysis procedure for computing predictability estimates for two ponds (SAL and CAM). **Two models are compared**: COL_ANa (discrete) and GAM_a (continuous). (a.1,2): Twenty-seven-year time series of the water-surface area (*A*) obtained by Landsat 5/7 scenes after applying the 2cA. (b.1,2): Counts for the two-way table (time steps vs. pond state) for the COL_ANa model; constancy, contingency and predictability indices are shown. (c.1,2): Scatter plot of the normalized water-surface area ($A’=A/\bar{A}$) and day of the year (DOY) from the complete time series; regression curves based on the GAM (solid line), 95% CIs (dashed lines), and predictability indices are shown.

Fig 5 illustrates how two models, one discrete (COL_ANa) and one continuous (GAM_a), estimate the predictability of the variability of *A* in the time series for two ponds, Salobralejo (SAL) and La Campana (CAM), which were selected due to their very different predictability estimations. Despite the reduction of information caused by the discrete classification or the regression model, the indices captured the different fluctuation patterns observed in the time series for each of the ponds.

The predictability estimates for each pond and model are shown in Table 3. Maximum predictability (i.e., 1) was achieved for three ponds (PET, SAL and SLD) with the COL_wd model because these ponds never completely dried out in our data record and the COL_wd model only considers the presence/absence of water. The coefficient of variation for the predictability estimations under each model ranged from 0.32 to 0.72, values that can be associated with the discrimination power of each model.

**Table 3:** Predictability estimates and statistics for each pond and model combination.

Pond	Model						
	COL_wd	GAM_a	COL_ANa	GAM_w	COL_ANw	COL_MAXg	COL_MAXlin
**PET**	1.00	2.23	0.66	2.28	0.64	0.48	0.26
**SAL**	1.00	3.03	0.82	3.07	0.86	0.39	0.55
**ONT**	0.97	2.04	0.62	2.08	0.67	0.17	0.26
**HYG**	0.32	0.74	0.22	1.14	0.12	0.41	0.42
**SLD**	1.00	2.21	0.80	2.26	0.77	0.34	0.21
**ATA**	0.75	2.27	0.67	2.43	0.74	0.48	0.32
**HOR**	0.23	1.02	0.29	1.91	0.56	0.34	0.34
**HYR**	0.66	2.07	0.66	2.35	0.72	0.48	0.30
**CVI**	0.13	0.73	0.31	1.62	0.51	0.34	0.44
**HRE**	0.17	0.54	0.39	1.90	0.52	0.45	0.53
**HYN**	0.18	0.58	0.35	1.46	0.35	0.53	0.67
**HYC**	0.12	0.83	0.21	1.61	0.45	0.23	0.33
**CAM**	0.11	0.64	0.16	1.02	0.20	0.41	0.52
**BLA**	0.63	1.94	0.55	2.13	0.71	0.37	0.24
**HMT**	0.19	0.72	0.28	1.58	0.35	0.39	0.47
**CVI2**	0.22	0.54	0.46	2.00	0.65	0.53	0.54
**HYA**	0.25	0.41	0.48	1.80	0.26	0.57	0.60
**HYB**	0.34	0.42	0.50	1.88	0.56	0.72	0.81
**HYE**	0.63	0.33	0.77	3.38	0.88	0.82	0.81
**HTU**	0.70	0.28	0.81	3.63	0.83	0.88	0.89
**Mean**	0.48	1.18	0.50	2.08	0.57	0.47	0.48
**Coefficient of variation**	0.70	0.72	0.43	0.32	0.39	0.48	0.43

**Fig 6:**
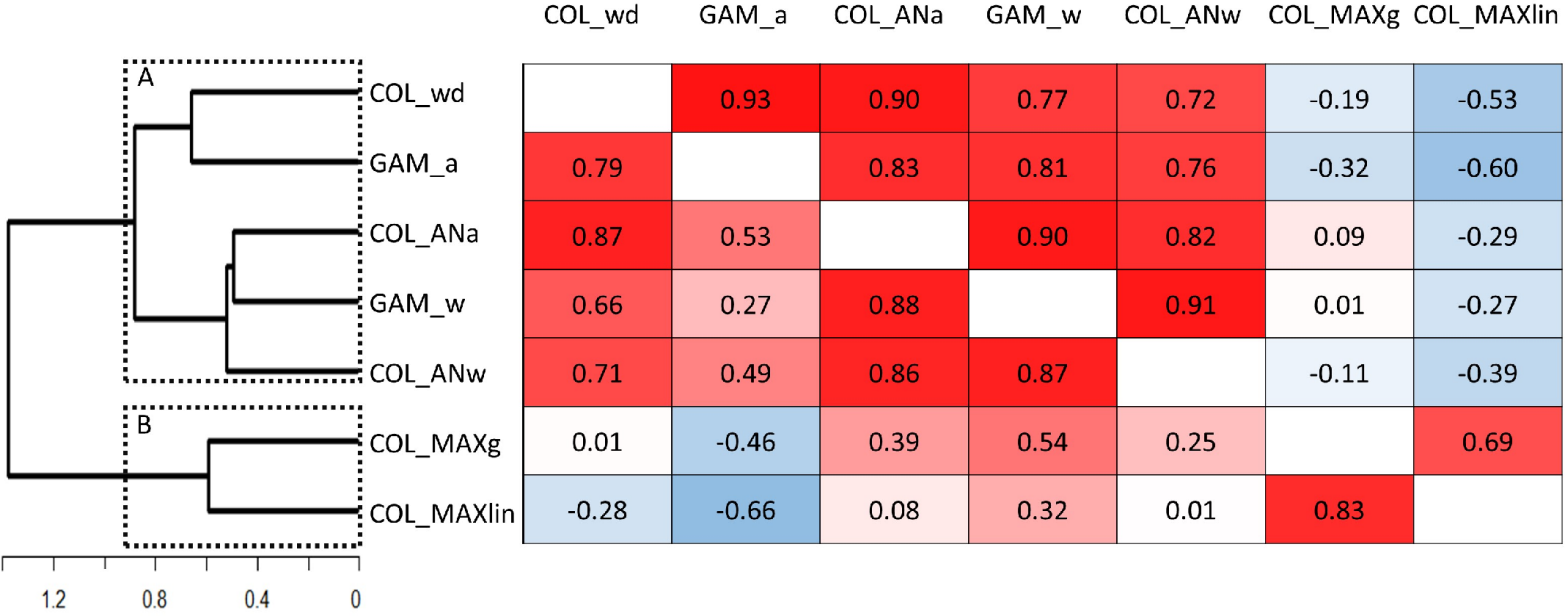
Relationships among the predictabilities estimated with the models. The matrix shows the correlations using Pearson’s coefficients obtained after excluding the two most ephemeral ponds (upper hemi-matrix, *n* = 18) and without excluding them (lower hemi-matrix, *n* = 20). The correlations are color-coded (blue-red scale, from negative to positive). A dendrogram obtained through a UPGMA cluster analysis based on the chord distance between predictability estimations (lower hemi-matrix) is shown. The two clusters of models were differentiated using 0.90 as the distance threshold, (A, and B; enclosed by dashed rectangles).

The relationships among models are shown in Fig 6. Two clusters of models were identified using 0.90 as the distance threshold. Cluster A includes the COL_wd, GAM_a, COL_ANa, GAM_w and COL_ANw models (bootstrap = 85%). This cluster includes discrete and continuous models. Moreover, the correlation between predictability estimates with discrete vs. continuous models ranged from 0.49 to 0.88. Thus, continuous and discrete approaches can produce similar results depending on other assumptions. The comparison of the two hemi-matrixes in Fig 6 showed that the correlation between GAM_a and the other models in Cluster A increased when excluding the two most ephemeral ponds (lowest correlation: 0.27 vs. 0.72).

**Fig 7:**
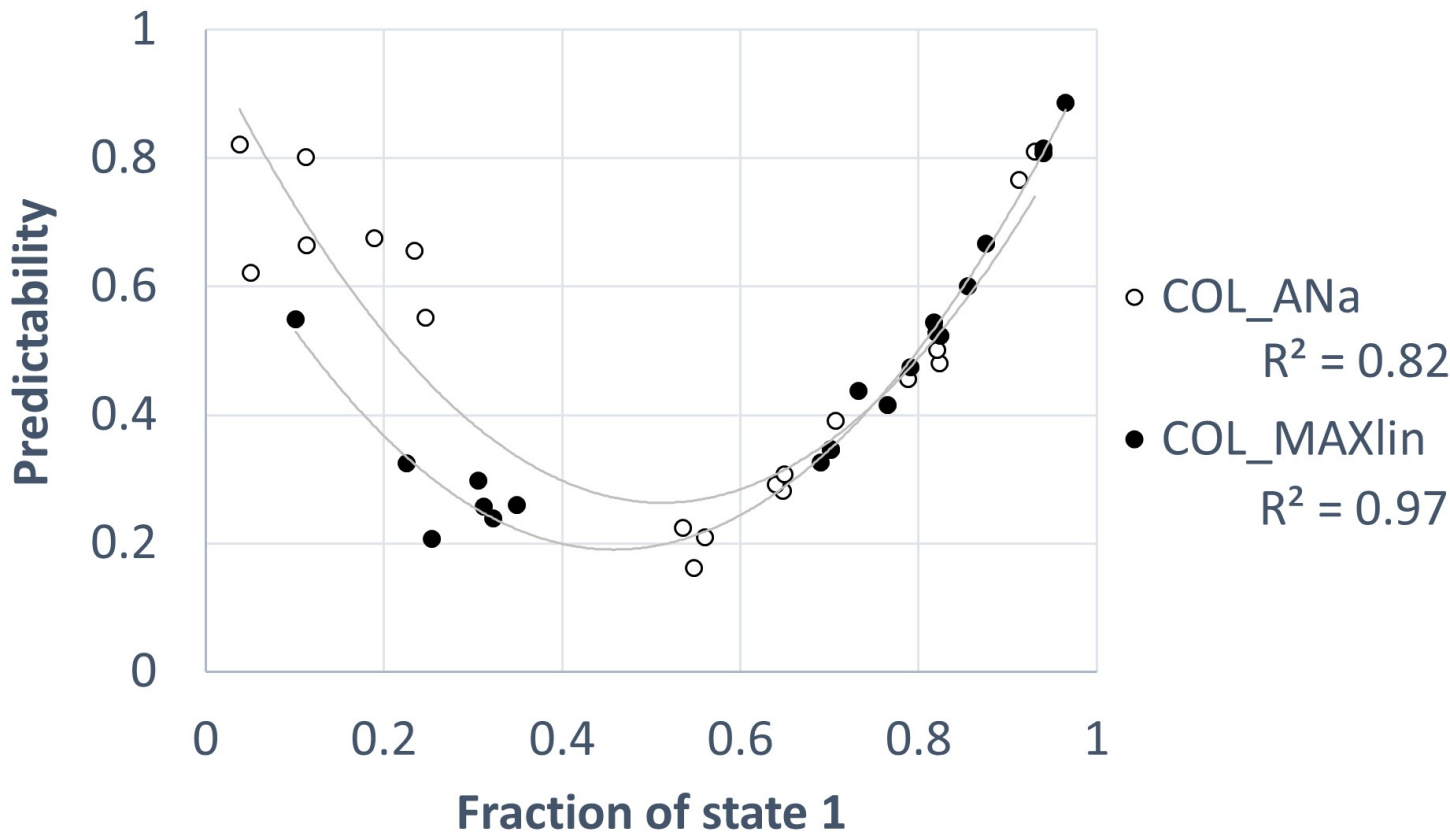
Relation between a model from each cluster (COL_ANa and COL_MAXlin) and the proportion of observations in state 1 (the state that includes *A* = 0). The solid line indicates the quadratic least-square fitting, and *R*^2^ is the determination coefficient associated with the corresponding fitting.

Cluster B includes COL_MAXg and COL_MAXlin (bootstrap = 99%), the two discrete models in which the states were defined with respect to the maximum observed *A*. Therefore, according to our results, linear and geometric scaling appears to be secondary for predictability estimations. Interestingly, some of the models in cluster A were negatively correlated with the models in cluster B. This diverging result is associated with the predictability index estimations in the ponds that frequently maintained some water cover (*A*>0), as shown in Fig 7. This figure depicts the performance of the models in clusters A and B, which was assessed by choosing a representative model from each cluster (COL_ANa and COL_MAXlin) and showing their predictability estimations against the proportion of observations in state 1 (the state that includes *A* = 0) for each pond.

## 5. Discussion

The characterization of environmental fluctuations is a central issue in ecology and environmental science that requires long-term time series [20]. Time series analysis often implies discarding a high volume of observations; in our case, 27 years of observations were reduced by 53% due to cloud filtering. Not surprisingly, obtaining long-term time series data is regarded as costly, and these datasets are consequently scarce [17]. In this context, remote sensing has been proven to be a timely, reliable, global and cost-efficient tool for analysing a given environmental variable over a long time series.

The general interest of our work relies first on the importance of evaluating long-term patterns of the water-surface area in ponds located in arid regions, where salt crust is a confounding feature in satellite images. These habitats are far from rare, and as dynamic island-like areas, their fluctuating patterns can be related to their biota (see below). The conditions for water detection applied in this study combine (1) the robustness of band ratios (MNDWI index>0 [40]) and (2) a refinement needed for saline ponds (near-infrared band reflectance<0.4, based on [42]). The consistency between the satellite-based measurements and measurements based on more direct observations was found to be notable. Our study shows that satellite scenes, after convenient processing, provide sufficient resolution for the detection of variations in *A* for saline ponds ranging from 0.00013 to 1.19 km^2^. We have shown that the pipeline established here allows quantification of the water cover in small ponds, which are of particular importance in ecological studies of arid regions.

Second, the general interest of our study relies on a conceptual analysis of the notion of predictability when it is quantitatively implemented using models. Based on our interest in practical applications, we performed our analysis using an actual system composed of time series for the water cover of 20 ponds over 27 years, which allowed us to identify divergences between predictability estimates. We also aimed to state that these differences among indices are related to how organisms perceive fluctuations in their environment. Thus, the choice of the predictability index should take into account the organism of interest.

One challenge is to assess the effect of the measurement scale (quantitative vs. qualitative) on the focal variable. Many studies need to be based on a nominal scale or just prefer this approach, while others use a quantitative scale. If quantitative data are available, a quantitative analysis appears *a priori* to retain more information than a qualitative one. Fourier analysis and AEMs [24,25] are alternative approaches for fluctuation analyses that address quantitative data but are unable to address aperiodic observations. Unfortunately, this case is not uncommon [62], and the continuous (GAM) models developed in this study address this problem. However, in our study, the cluster analysis merges predictability estimates from discrete (nominal) and continuous (quantitative) models. A direct inspection of correlation values among predictability estimates confirmed that the cluster analysis does not force the aggrupation of some discrete and some continuous models. Therefore, these approaches can yield similar predictability results, and other assumptions have a higher impact on predictability estimates. Nevertheless, in contrast with Fourier analysis, both approaches (Colwell’s approach and the GAMs) ignore the correlation between consecutive observations because the days of the year in different years are merged, which makes them share an underlying assumption that might account for the similar estimations found in this study. Further studies should develop a time-series analysis procedure that takes into account the correlation between consecutive observations and is able to address rather aperiodic observations. In the meantime Colwell approach, which involves the discretization of a continuous variable, appears to be reliable compared with the regression methods explored in this study and has the advantage of keeping the analysis simple [20, 63, 64]. However, the discretization criteria could critically affect the predictability estimations, and that is needed to be closely looked by researchers intending to apply Colwell’s approach (see below).

Thus, a second challenge is to determine the type of variation in data that is relevant for estimating unpredictability. For instance, extreme values could be much more–or much less–important than would be accounted for in a linear approach, and unpredictability estimations could be inflated by considering meaningless values. This problem translates into the scale transformation (e.g., normalization) applied to the focal variable, which cannot be thoroughly analysed without considering the organism for which unpredictability is evaluated. In previous stream and river studies, the presence of many zeros in the time series caused the non-zero observations to produce an extreme residual [20]. Here, we explored different models that consider or not consider zeros in several ways, and we obtained similar results in the majority of cases. However, one of the continuous models (GAM_a) differed from the rest of the models in terms of classifying the two most ephemeral ponds (HTU and HYE; *A* = 0 in more than 90% of observations), which likely caused the low correlation between GAM_a and some of the models in cluster A. Most of the models assigned a high degree of predictability to these two ponds, whereas GAM_a assigned them a low degree of predictability. Predictability estimation by GAM_a is based on the coefficient of variation of *A* and tended to be high for these two ponds (thus yielding low predictability) because their mean *A* is very low. This effect on the coefficient of variation has been reported in statistical studies [65, 66]. In other words, GAM_a–and more generally, the consideration of zeros to estimate the mean *A* in a continuous approach–could inflate the estimation of environmental unpredictability. This effect is supported by direct inspection of the data. In fact, HTU and HYE ponds rather than unpredictable systems appear to be ephemeral ponds, as they are dry most of the time (i.e., predictably dry).

As has been stressed for many years [8,9], predictability depends on the point of view of the organism of interest, and confusion is possible between what human researchers and what other organisms can predict. Therefore, no predictability metric can pretend to serve as an absolute quantification of the degree of predictability of a specific environment, but instead, time scales of periodic variations and other biological factors must be taken into account when referring to any specific predictability estimation [10]. Models designed under different biological assumptions are expected to produce different predictability results, which is the case when comparing models between each of the two identified clusters. The organism’s features that affect its capability to predict fluctuations are potentially numerous and poorly known; thus, the proposal of general rules for selecting an appropriate predictability metric is inherently difficult. In contrast, performing a selection based on the biological information available for the case of interest is advisable. For instance, an inspection of the model assumptions suggests that predictability in the models in cluster A is the one perceived by a generalist, eurioic, small-sized organism, whereas the opposite is true for the models in cluster B. Hence, in model COL_wd (cluster A), the presence of water–either with high *A* or low *A*–would be sufficient to make the environment suitable. Consistently, in COL_ANa and COL_ANw, a wide variation around the mean *A* value does not result in a state change. Thus, taking into account the case of animals, the scaling of the models in cluster A could fit the case of aquatic invertebrates (such as cladocerans, rotifers, copepods and insect larvae, i.e., organisms sized less than ca. 1 cm) that can achieve high population sizes in small volumes and tolerate a broad variation in *A*. This *a priori* assessment was confirmed after dissecting the outputs from the models. As shown in Fig 7, the models in both cluster A and cluster B did not confound ponds that are frequently dry with unpredictable ponds. Both ephemeral ponds and nearly permanent ponds achieved high predictability estimations. Thus, (a) frequent maintenance of some water and (b) frequently dry or with a low water cover can be regarded as predictable for these animals. In contrast to cluster A models, the models in cluster B assign a lower predictability to the environment of ponds that frequently maintain water. This finding can be interpreted as that the models in cluster B are sensitive to random variations in the amount of water when water occurs. This sensitivity is welcomed if the organism of interest is affected by the extension of the water cover, not being sufficient condition the presence of some water. This is likely the case for large animals that typically need a large exploitation area and are close to the top of the trophic chains (e.g., fish). Our ponds offer few opportunities for these animals because, according to our analysis, the studied ponds do not fit in such environments. Consistently, the animals typically found in our study area are mostly restricted to small invertebrates [60,67] that are able to display diapause stages. To the best of our knowledge, no large animals have been reported in our study system, with the exception of migratory birds [68]. For waterfowls, the seasonal home range would be the whole pond system rather than a single pond because they can move easily from one pond to another. Therefore, if the fluctuation of *A* is poorly coupled among ponds–as found in our system–waterfowls could exploit the ponds with high *A*, making the system more predictable for them. In other words, single-pond fluctuation is fine-grained for waterfowls.

## 6. Conclusions

When characterizing fluctuations in a natural system, ecologists face the following two problems: the need to gather long-term data and the handling of these valuable data that are tough to acquire. We have shown that remote sensing data have become more accessible, which opens an opportunity for ecologists to obtain the long time series needed to calculate predictability metrics. Our analysis at this step is restricted to a specific variable (*A*) and type of habitat, but this type is important in many geographic regions. Once this long-term data series is acquired, a model that produces a predictability metric has to be developed. We thus performed a conceptual analysis in which we propose several models based on how the variation of the focal variable can be relevant to different organisms with different strategies and life histories. Interestingly, in addition to Colwell’s approach, we have introduced GAM fitting as an alternative approach for measuring predictability; thus, the effect of a continuous vs. a discrete approach could be studied. Our methodology extracted meaningful information regarding the degree of predictability of a set of Mediterranean ponds. Interestingly, some model assumptions have been shown to exert minor effects (as shown by the correlation data and the cluster analysis). In contrast, other model assumptions caused divergences in predictability estimations, and we propose that this divergence can be associated with the differences in how the organism of interest perceives fluctuations in its environment. The methodology described in this manuscript is applicable to a wide variety of study systems and will be valuable for quantifying and characterizing predictability, which is essential considering the predicted scenario of upcoming global increases in the unpredictability of environmental fluctuations [3,5]. Interestingly, the satellite Sentinel 2A, which was recently launched in 2015, and satellite Sentinel 2B, which will be lunched in the near future, will work with Landsat 8 to provide images every three to five days. This improvement in the temporal resolution of remote sensing data will enable us to improve the area estimations and better study the variability.

## Supporting information

S1 Fig Relationships among the water-surface area (*A)* obtained through a direct inspection of aerial scenes and the *A* values from raw satellite scenes after applying only the first condition (left panel, Y axis) and after applying the 2cA assessment (right panel, Y axis). The dots are the values for 19 out of 20 ponds recorded in the years 2006 and 2009. The maximum time for matching values corresponding to the two estimations was 18 days. Linear least squares fitting is represented as a continuous line, and the corresponding equation and determination coefficient are shown in the upper right corner of each panel. The dashed line represents the ideal situation in which a perfect match exists between the aerial and satellite estimations of *A*. *n* = 33; the pair 0–0 was found in 18 cases in the left panel and 22 cases in the left panel.
(DOCX)

S2 Fig Bars: Percentage of satellite scenes in which salt-covered pixels (TM4< 0.4) were detected after retaining potentially water-covered pixels (MNDWI> 0) at each pond. The means and standard deviations (shown between parentheses) of the percentage of reduction are shown above.
(DOCX)

S1 Table Data used for validation of satellite water-surface area estimation (from aerial scenes) and presence/absence of water (from field observations). (DOCX)

S2 Table Number of pixels assigned as water–covered pixels and to cloud-covered pixels in each pond (columns) and satellite scene (rows). (XLSX)
