# Supplementary material for: Quantifying unpredictability: A multiple-model approach based on satellite imagery data from Mediterranean ponds
Source: PLoS One. 2017 Nov 9;12(11):e0187958. doi: 10.1371/journal.pone.0187958 (PMC5679618; doi:10.1371/journal.pone.0187958)
Supplement: S1 Fig — The dots are the values for 19 out of 20 ponds recorded in the years 2006 and 2009. The maximum time for matching values corresponding to the two estimations was 18 days. Linear least squares fitting is represented as a continuous line, and the corresponding equation and determination coefficient are shown in the upper right corner of each panel. The dashed line represents the ideal situation in which a perfect match exists between the aerial and satellite estimations of A. n = 33; the pair 0–0 was found in 18 cases in the left panel and 22 cases in the left panel. (DOCX) [file pone.0187958.s001.docx]

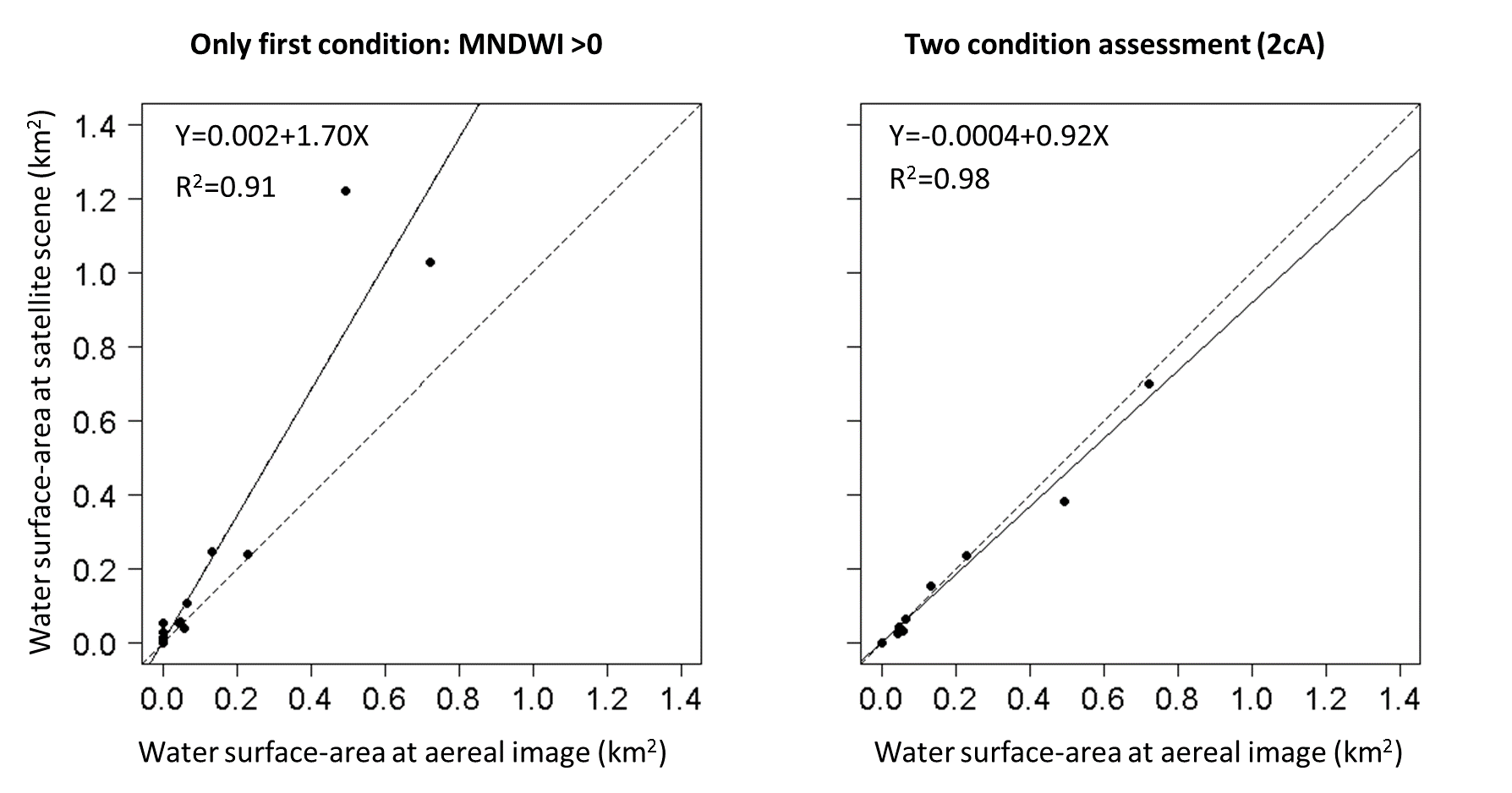


**S1 Fig. Relationships among the water-surface area (*A)* obtained through a direct inspection of aerial scenes and the *A* values from raw satellite scenes after applying only the first condition (left panel, Y axis) and after applying the 2cA assessment (right panel, Y axis).**  The dots are the values for 19 out of 20 ponds recorded in the years 2006 and 2009. The maximum time for matching values corresponding to the two estimations was 18 days. Linear least squares fitting is represented as a continuous line, and the corresponding equation and determination coefficient are shown in the upper right corner of each panel. The dashed line represents the ideal situation in which a perfect match exists between the aerial and satellite estimations of *A*. *n*=33; the pair 0-0 was found in 18 cases in the left panel and 22 cases in the left panel.
