# Supplementary material for: Quantifying unpredictability: A multiple-model approach based on satellite imagery data from Mediterranean ponds
Source: PLoS One. 2017 Nov 9;12(11):e0187958. doi: 10.1371/journal.pone.0187958 (PMC5679618; doi:10.1371/journal.pone.0187958)
Supplement: S2 Fig — The means and standard deviations (shown between parentheses) of the percentage of reduction are shown above. (DOCX) [file pone.0187958.s002.docx]

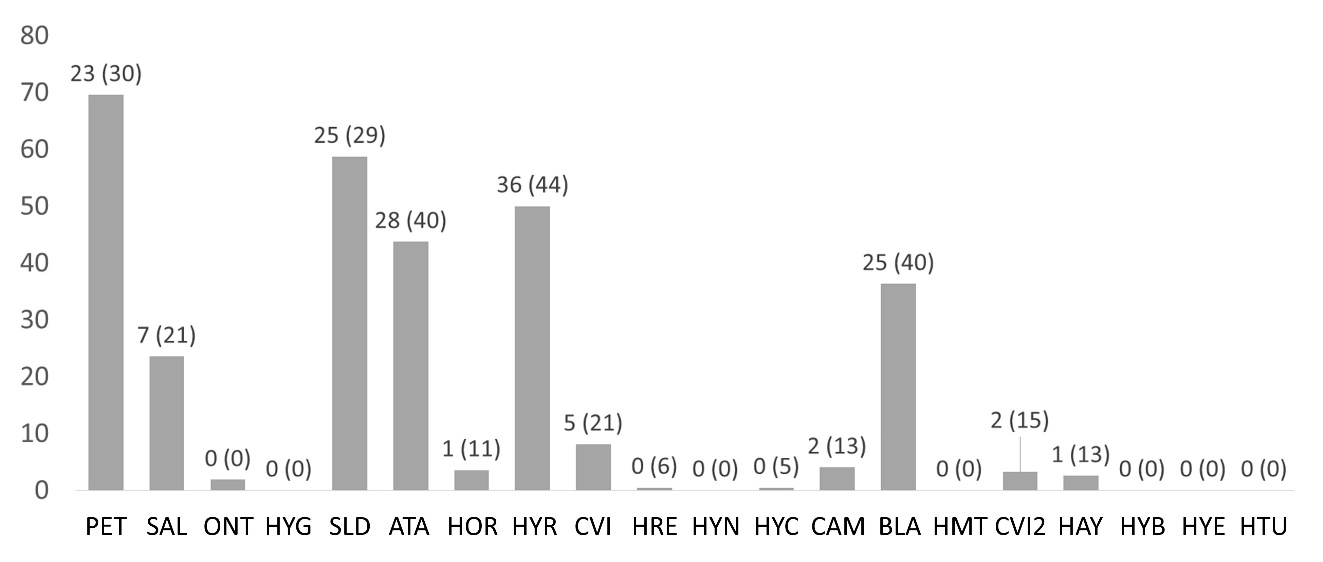


**S2 Fig. Bars: percentage of satellite scenes where salt-covered pixels (TM4< 0.4) were detected after retaining potentially water covered pixels (MNDWI> 0) at each pond**. The mean and standard deviation (the latter between parentheses) of the percentage of reduction are shown above
