# Supplementary material for: Quantifying unpredictability: A multiple-model approach based on satellite imagery data from Mediterranean ponds
Source: PLoS One. 2017 Nov 9;12(11):e0187958. doi: 10.1371/journal.pone.0187958 (PMC5679618; doi:10.1371/journal.pone.0187958)
Supplement: S1 Table — (DOCX) [file pone.0187958.s003.docx]

**S1 Table. Data used for validation of satellite water-surface area estimation (from aerial scenes) and presence/absence of water (from field observations).**

| Date | Source | Ponds |
| --- | --- | --- |
| May 2000 | Field | PET;SAL |
| June 2000 | Field | PET;SAL |
| July 2000 | Field | PET;SAL |
| August 2000 | Field | PET;SAL |
| September 2000 | Field | PET;SAL |
| October 2000 | Field | PET;SAL |
| November 2000 | Field | PET;SAL |
| December 2000 | Field | PET |
| January 2001 | Field | PET;SAL |
| March 2001 | Field | PET;SAL |
| April 2001 | Field | PET;SAL |
| July 2001 | Field | PET;SAL;HYR |
| November 2001 | Field | PET;SAL;HYR |
| July 2002 | Field | PET |
| September 2004 | Field | SAL |
| October 2004 | Field | PET;SAL;HYR;HYM |
| November 2004 | Field | PET |
| January 2005 | Field | SAL |
| February 2005 | Field | PET;SAL |
| March 2005 | Field | PET;SAL |
| April 2005 | Field | PET;SAL |
| May 2005 | Field | PET;SAL |
| June 2005 | Field | PET;HYR |
| August 2005 | Field | PET |
| September 2005 | Field | PET |
| February 2006 | Field | PET |
| March 2006 | Field | PET;SAL |
| 14/07/2006 - 2/08/2006 | Aerial | PET;SAL;HYG;SLD;ATA;HOR;HYR;CVI;HRE;HYN;  HYC;CAM;BLA;HMT;CVI2;HYA;HYB;HYE;HTU |
| 16/07/2009 - 25/07/2009 | Aerial | PET;SAL;HYG;SLD;ATA; HYR; HRE;HYN;  CAM;BLA;HMT;HYB;HYE;HTU |
| July 2010 | Field | PET;SAL |
